# Friction Stir Welding/Processing of Mg-Based Alloys: A Critical Review on Advancements and Challenges

**DOI:** 10.3390/ma14216726

**Published:** 2021-11-08

**Authors:** Farzad Badkoobeh, Hossein Mostaan, Mahdi Rafiei, Hamid Reza Bakhsheshi-Rad, Filippo Berto

**Affiliations:** 1 School of Metallurgy and Materials Engineering, College of Engineering, University of Tehran, Tehran, Iran; farzad.badkoobeh@ut.ac.ir; 2 Department of Metallurgy and Materials Engineering, Faculty of Engineering, Arak University, Arak, Iran; 3 Advanced Materials Research Center, Department of Materials Engineering, Najafabad Branch, Islamic Azad University, Najafabad, Iran; 4 Department of Mechanical and Industrial Engineering, Norwegian University of Science and Technology, 7491 Trondheim, Norway

**Keywords:** magnesium-based alloys, friction stir welding, friction stir processing, severe plastic deformation, microstructure, texture, mechanical properties

## Abstract

Friction stir welding (FSW) and friction stir processing (FSP) are two of the most widely used solid-state welding techniques for magnesium (Mg) and magnesium alloys. Mg-based alloys are widely used in the railway, aerospace, nuclear, and marine industries, among others. Their primary advantage is their high strength-to-weight ratio and usefulness as a structural material. Due to their properties, it is difficult to weld using traditional gas- or electric-based processes; however, FSW and FSP work very well for Mg and its alloys. Recently, extensive studies have been carried out on FSW and FSP of Mg-based alloys. This paper reviews the context of future areas and existing constraints for FSW/FSP. In addition, in this review article, in connection with the FSW and FSP of Mg alloys, research advancement; the influencing parameters and their influence on weld characteristics; applications; and evolution related to the microstructure, substructure, texture and phase formations as well as mechanical properties were considered. The mechanisms underlying the joining and grain refinement during FSW/FSP of Mg alloys-based alloys are discussed. Moreover, this review paper can provide valuable and vital information regarding the FSW and FSP of these alloys for different sectors of relevant industries.

## 1. Introduction

Magnesium (Mg)-based alloys are widely used in the industries of automotive, aerospace, electronics, transportation, and so on [1,2,3,4,5]. The cause of this is related to low density (i.e., low weight), high shock absorption, high damping, good electromagnetic shielding, high specific strength, and good hot formability [2,3,4,6,7]. On the other hand, the casting of these alloys is cost-effective, and they can be recycled [1,2,7,8]. Mg alloys are also used in the nuclear industries due to their low tendency to absorb neutrons, excellent resistance to carbon dioxide, and suitable thermal conductivity [2]. In addition, Mg alloys are applicable as a biomaterial caused by their desirable biocompatibility [9,10,11,12,13,14]. Unfortunately, the formability behavior and ductility of Mg alloys are undesirable at room temperature, resulting in premature failure and potentially severe failure under complicated stress circumstances. This is due to the presence of a strong basal texture, the limitation of the number of active slip systems in the hexagonal close-packed (HCP) crystal lattice, and their low symmetry [2,15,16,17]. These reasons cause limited strength, low fatigue resistance, and low creep resistance [2]. According to the assessments, undesirable formability of the as-cast Mg alloys has been caused by coarse grains, the presence of the interdendritic micro-porosities, and the creation of coarse eutectic micro-constituents as well [15]. It is suggested that the behavior of the Mg alloys is considerably modified via the addition of rare-earth (RE) elements such as Y, Nd, etc. It was also concluded that the interdendritic long-period stacking ordered (LPSO) phases could be created as a block shape with the addition RE elements. On the other hand, adding Zn element to Mg-RE alloys can effectively form LPSO phases [8,18,19]. Basically, the addition of RE elements to Mg alloys results in activation of non-basal slip systems, grains refining, and weakening the strong basal texture [4,18,19,20]. It is quite obvious that grain refinement results in an improvement in strength and toughness simultaneously. Accordingly, the best method for increasing them as well as formability in Mg-based alloys is to make grain refinement and randomized basal texture (i.e., modification of strong basal texture) [6].

The primary methods for enhancing the mechanical performance of magnesium alloys include alloy composition, recrystallization, and grain refining. By refining the grains and regulating the texture, plastic deformation can improve the mechanical performance of alloys [21]. Nowadays, severe plastic deformation (SPD) methods are used to create a fine-grained, ultrafine-grained, and nano-grained microstructure [22,23]. These methods consist of equal-channel angular pressing (ECAP), high-pressure torsion (HPT), accumulative roll bonding (ARB), differential speed rolling (DSR), friction stir processing (FSP), etc. [15,24,25]. Among them, FSP is a very efficient and useful method because the tensile strength of the stir zone (SZ) does not drop, and this zone is ductile [15,25,26,27]. FSP is a solid-state SPD with a high strain rate that originates from friction stir welding (FSW) [27,28,29,30,31]. The mechanical properties of the fusion joints in Mg alloys are undesirable. The formation of coarse grains, brittle intermetallic compounds, cracks, cavities, and oxidized layers are only some drawbacks of fusion joints [2,8]. While FSW is a solid-state joining technique and these problems can be prevented [2,8,27,28]. In FSP, the heat required for plastic deformation of a material is provided through generated frictional heat between the rotating tool and the base metal and heat-related to the plastic deformation of the base metal. Thus, FSW/FSP treatment does not need the material to be pre-heated, and it is carried out at room temperature [15,27,28,29,32]. Generally, by performing FSW/FSP, the microstructure and texture of the material are modified without decreasing the fracture strength [2,6,27]. FSW/FSP causes a grain refinement in SZ, and on the other hand, a uniform microstructure is obtained [14,23,27,28,33,34,35,36]. Furthermore, it might be done locally on a structural component’s targeted area without affecting the shape of the metal components [37]. Another important effect of FSW/FSP is texture softening, and even texture strengthening can take place [19]. Moreover, FSW/FSP changes the distribution of secondary phases [27,38]. These can be attributed to the occurrence of SPD at high temperatures obtained by producing the frictional heat, followed by dynamic recrystallization (DRX) during these techniques [27,28,32,39,40,41,42,43]. As a result, it can be said that the formability is significantly affected by FSW/FSP and can be improved by these processes [27,28,32,39,40,41,42,43]. It is essential to say that mechanisms of DRX are variant and can be discontinued DRX (DDRX), continued DRX (CDRX), geometric DRX (GDRX), twin DRX (TDRX), or particle-stimulated nucleation (PSN) depending on conditions [27,28,44]. It is worth noting that the brittle eutectic phases are reduced by FSW/FSP, which is associated with massive heat input due to the stirring process [38]. Meanwhile, the high-temperature SPD created by FSW/FSP causes dissolution of eutectics, precipitations refinement, and reduction of segregation of solute atoms in the as-cast Mg alloys [45]. These phenomena can enhance the formability of Mg alloys [38,45]. Fine-grained, ultrafine-grained, and even nano-crystalline structures might be created by FSW/FSP [27,38]. Whereas FSP outperforms other traditional processing approaches, it does have a disadvantage. When FSP was used, a significant amount of plasticity was attained. Nevertheless, because of the unavoidable texture alteration produced by FSP, a rapid decline in yield stress, along with flow stress, was always present. This might be regarded as the negative side of using the FSP approach on Mg-based alloys [37,46,47]. Even though the material might be further strengthened by reducing the grain size to the submicron level, the material’s flexibility usually suffers as a result. The question then becomes whether it is feasible to consolidate the benefits of FSP with other types of SPD approaches, including surface mechanical attrition treatment (SMAT), and compensate shortcomings each other in order to get an optimal total mechanical performance in Mg-based alloys [37,48]. Similarly, Lee et al. [49] have also shown that further compressive loading in the vertical position of FSPed AZ61 alloy might escalate the yield strength of the treated specimens. Similar results were suggested regarding subsequent straining of FSPed Mg alloy via rolling [50], tensile loading, and bending [51,52]. It is well known that combining FSP with post-treatment to improve the mechanical characteristics of Mg alloys is a simple and successful method [53]. It is considered that the Mg alloys are extremely sensitive to heat than Al alloys, which resulted in the complication of the treatment. Hence, FSP of Mg alloys is performed with the implementation of an additional cooling system intended for structure refinement. In this regard, Chang et al. [23,54] employed liquid nitrogen-cooled FSP to fabricate AZ31 alloy with a fine-grained structure [55], while Yuan et al. [56] employed a copper backing plate to escalate the cooling rate throughout FSP of AZ31 alloy, presenting the UFG with formidable basal texture, which has a significant effect on mechanical performance [55]. It has been shown that nano-grains can be formed by the supercooling path [6,57,58,59]. Chai et al. [60] examined the microstructure and superplastic tensile performance of as-cast AZ91 plate conventional FSP (treated in the air) and immersed FSP (treated in water, SFSP) alloys. The increased cooling rate of SFSP results in impressive grain refinement when compared to standard FSP, with an average grain size of 1.2 mm and 7.8 mm. In comparison to the regular FSP specimen, the SFSP AZ91 specimen has significantly improved superplastic ductility, lower flow stress, and a greater optimal strain rate. The basic mechanism for superplastic deformation of typical FSP and SFSP materials is grain boundary sliding. It was shown [25,61,62,63] that the FSWed or FSPed material can acquire the capability of superplasticity because of the formation of a fine-grained or ultrafine-grained structure during FSW/FSP. Liu et al. [64] demonstrated that FSP could lead to considerable grain refinement and microstructure uniformity in the SZ of the AZ31 alloy owing to complete DRX. Due to grain refinement and subgrain boundaries, FSP can enhance the mean microhardness of the SZ to some extent so that in this material, grain boundary sliding (GBS) becomes the dominant mechanism of plastic deformation at high temperatures and high strain rates during superplastic deformation [25,65,66]. In this way, the necking phenomenon delays during tensile loading and ultra-high elongations can be obtained [25,61,65,67]. Taking into account the importance of described issues, the main objective of this review article is to present grain refinement mechanisms during the FSP and FSW techniques. The review also presents valuable information for better understanding the relationship between microstructure evolution and mechanical characteristics of the FSW/FSPed Mg alloys to improve the properties of surface-treated specimens.

## 2. Research Advancement concerning FSW/FSP of Mg-Based Alloys

As mentioned earlier, Mg alloys have been attracted great attention in various industries due to their excellent properties. FSW and FSP techniques also are attractive processes in the same industries [2,27,28]. Accordingly, the accomplishment of FSW/FSP in these alloys is definitely appealing and urgent. Moreover, the dissimilar FSW of Mg alloys is also used extensively. It is reported [68,69,70] that FSW is now deemed developed enough to be used in aircraft manufacture, and its capacity to combine dissimilar metals in different configurations has already been proved. FSW, among other welding methods, reduces metallurgical flaws and distortion while also reducing weight and expense. Meanwhile, other studies [71,72,73,74] pointed out that the quality of the joints, which is mainly associated with processing factors, affects the mechanical characteristics of welded structures. Consequently, several investigations have been implemented on these subjects. Hence, a summary of the works performed on Mg-based alloys subjected to FSW/FSP is presented in Table 1. Based on Table 1, the FSW/FSP of Mg alloys is extremely affected by specific parameters.

## 3. Joining Mechanism of FSW/FSP of Mg-Based Alloys

**Figure 1:**
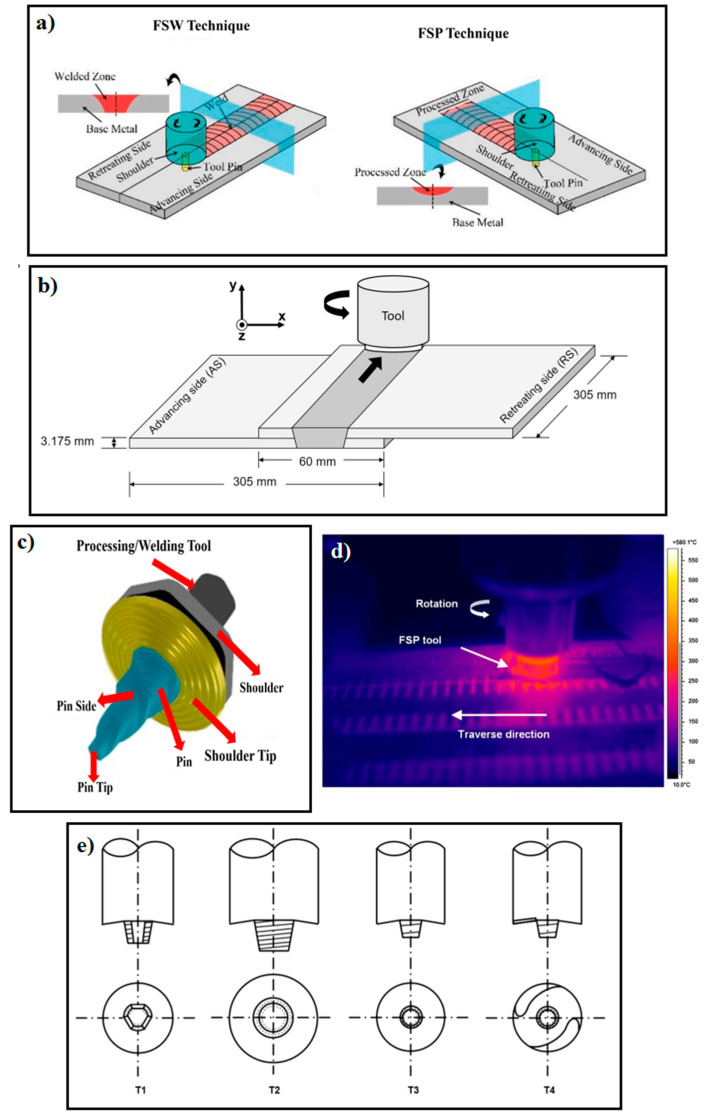
(**a**) Schematic representation of FSW technique, and FSP technique (Adapted from [27]). (**b**) Lap joint configuration of the 2024-T3 AlClad FSW assembly (Reprinted with permission from ref. [10]. Copyright 2020 Elsevier Ltd.). (**c**) Rotation tool in FSW/FSP technique (Adapted from [27]). (**d**) IR image of FSP tool during processing (Reprinted with permission from ref. [7]. Copyright 2016 Elsevier B.V.). (**e**) Different FSP tool pin profiles (Reprinted with permission from ref. [7]. Copyright 2016 Elsevier B.V.).

**Figure 2:**
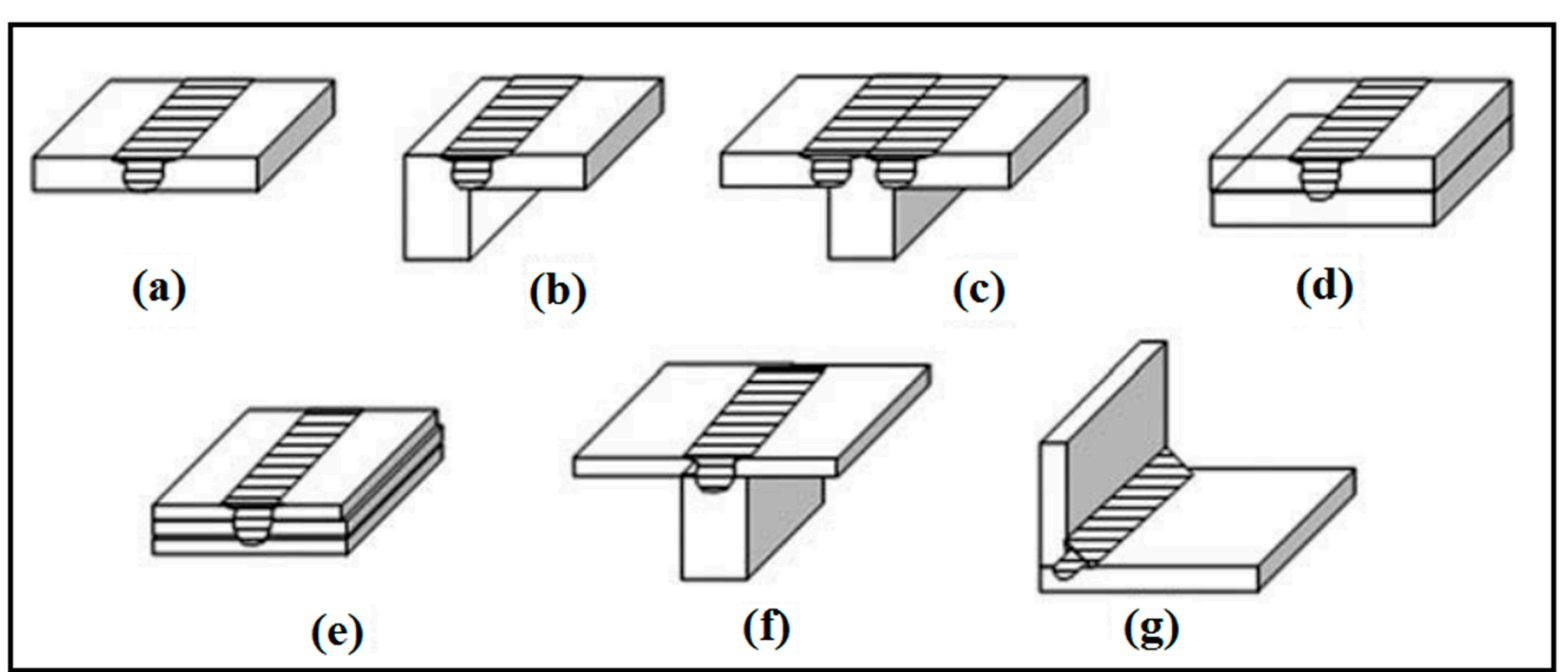
Joint configurations for friction stir welding: (**a**) square butt, (**b**) edge butt, (**c**) T butt joint, (**d**) lap joint, (**e**) multiple lap joint, (**f**) T lap joint, and (**g**) fillet joint (Reprinted with permission from ref. [91]. Copyright 2005 Elsevier B.V.).

A schematic representation of FSW and FSP can be observed in Figure 1a [27]. In the FSW technique and the lap joint design, according to Figure 1b, two materials/plates lapped should be firstly clamped on a backing plate (support) [10]. Then, a rotary tool consisting of a pin and shoulder is used, as seen in Figure 1c [27]. Rotary tools are usually made of tool steels, hot work, and high-speed steels. This rotary tool that is non-consumption is vertically plunged across the top plate into the bottom plate with very high speed and goes through along the desired direction [2,27,28]. However, in the butt joint design, two plates to be joined to each other are firstly fixed together by a fixture. Then, the rotary tool is plunged into the joint line and traverses along the joint line [2,27,28,89,90]. The highest temperature listed for the plate surface close to the tool path regarding the various tools was tested by using a ThermaCAM S40 infrared camera. An infrared image taken throughout the FSP process is presented in Figure 1d [7]. In general, it should be noted that the shoulder is placed on the surface, and the pin is partially plunged into the two materials to be joined. The pin also applies a uniaxial compressive force on the surface and travels along the desired direction by rotating the tool. The tool is plunged into the joint line and travels along the joint line [2,27,28]. The forces are large enough during the primary plunging of the rotary tool and must be assured of the position of the joint. The rotation of the tool and the pin movement accompanied by its compressive force on the surface cause a generation of frictional heat in the interface of the tool shoulder and workpiece. Via the frictional heat created, the material is softened at the joint place. Hence, this softened material is stirred by the rotating pin and flows circularly [2,27,28]. Generally, the pin controls the act of stirring. The flow of the material is also similar to the extrusion process where the cavity produced is filled back to the tool. Accordingly, two materials are blended, and a joint is created in their interface. It should be mentioned that the frictional heat generated results in increasing temperature, but it does not reach the melting point of the material [2,27,28]. The zones produced in the material structure after the FSW will also be discussed in detail. To achieve a suitable joint, the material must flow at high temperatures. As the temperature increases, the flow stress decreases. More and faster reduction of flow stress with temperature will facilitate FSW [2,27,28]. It is important to state that the geometry of the tool is also of great importance since the tool plays a key role in localizing the heat and the material flow [2,27,28]. Shoulder shape and its surface characteristics, the tool probes (or pin), and the tools designed in The Welding Institute (TWI) are seen in Figure 1e [7]. The most common joint designs in FSW are lap and butt. However, there are other joint designs, as illustrated in Figure 2 [91]. These include: (a) square butt, (b) edge butt, (c) T butt joint, (d) lap joint, (e) multiple lap joint, (f) T lap joint, and (g) fillet joint [91].

**Figure 3:**
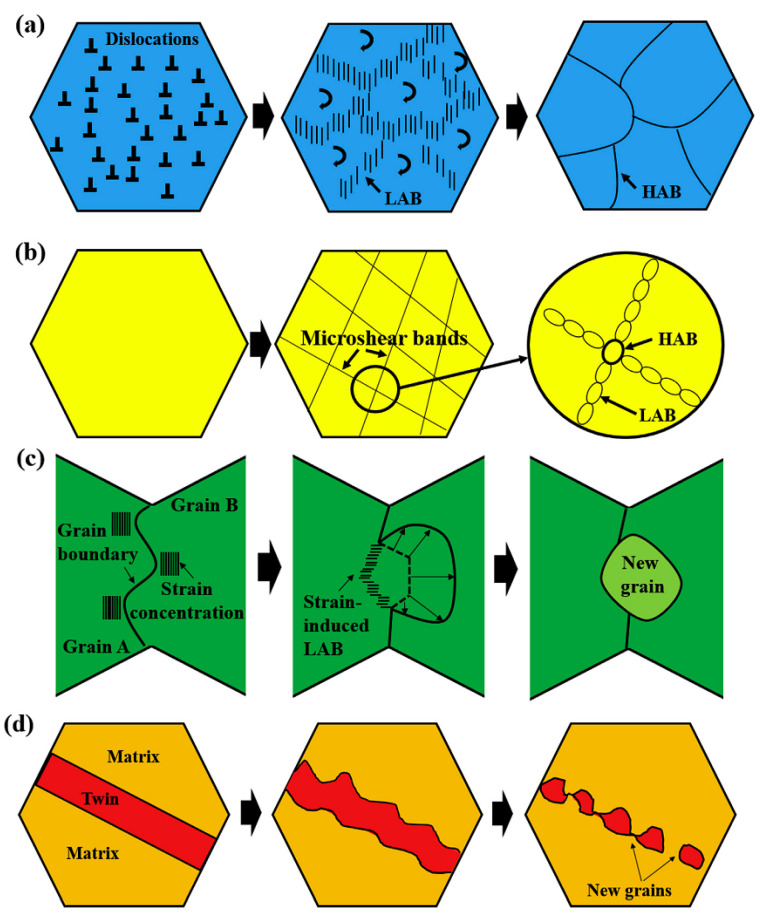
Schematics of several grain refinement mechanisms during the CSA-FSP of the AZ31B magnesium alloy including (**a**) strain-induced CDRX, (**b**) micro-shear band induced CDRX, (**c**) strain-induced DDRX, and (**d**) twinning-induced DRX (Reprinted with permission from ref. [57]. Copyright 2019 Elsevier B.V.).

FSP is an advancement of the FSW technique, as shown in Figure 1a. However, no joints will be established in FSP, and the FSP purpose is not welding [2,27,28]. The FSP mechanism is exactly the same as the FSW [92,93] so that the rotating tool (see Figure 1c) moves in a straight direction in the FSP (usually along the longitudinal direction of the workpiece); then, the tool pin creates a cavity that is filled by the softening flow of the material. In other words, the material surface is processed by the FSP; hence, this technique is employed for surface modification of the materials [2,27,28,94]. It is noteworthy that these technologies, i.e., FSP and FSW, are such important and usable methods for the production of metal matrix composites (MMC) [27,95,96,97]. This means that the secondary phase particles can be merged with the material. In this way, at first, a groove or several cavities are created on the surface of the workpiece. Then, the secondary phase particles (i.e., reinforcement) are poured into the grooves. Finally, the particles are combined with the workpiece by rotating the tool, and a composite structure is developed [27,95,96]. Reinforcements can consist of ceramics such as nitrides, oxides, carbides, and other materials [96]. Nano-sized reinforcements, such as the mentioned ceramics and particularly carbon nanotube (CNT) and graphene, can make excellent properties [96]. In this regard, Vahedi et al. [98] used the FSP approach to fabricate AZ31/Gr and AZ31/GNPs composites, and their findings revealed that the grain size of Mg reduced from 40.65 at an original position to 5.63 and 2.29 mm for AZ31/Gr and AZ31/GNPs composites, respectively, owing to the Zener restricting grain size and the pinning impact of nanofillers on grain boundaries. Xu et al. [57] conducted FSP with a cooling system on AZ31B alloy to refine the structure and enhance mechanical characteristics. Their finding revealed that liquid CO_2_ has high cooling effectiveness, preventing grain coarsening and stimulating the {10–12} twinning characteristic in the surface region, which leads to fabricating UFG structures with numerous twin boundaries. The schematic of grain refinement mechanisms is presented in Figure 3 [57]. In general, the ratio of low angle boundaries (LAB) in the transition zone (TZ) is typically greater than in the SZ. Non-equilibrium grain boundaries are caused by strain-induced continuous DRX (CDRX)/discontinuous DRX (DDRX), and micro-shear band generated CDRX, which result in high dislocation density in the grain interior and uneven grain boundaries with abundant dislocations [58,59]. Hence, the primary grain refinement mechanisms during the cold source assistant FSP (CSA-FSP) of the AZ31B magnesium alloy are strain-induced CDRX/DDRX and micro-shear band induced CDRX [57].

**Figure 4:**
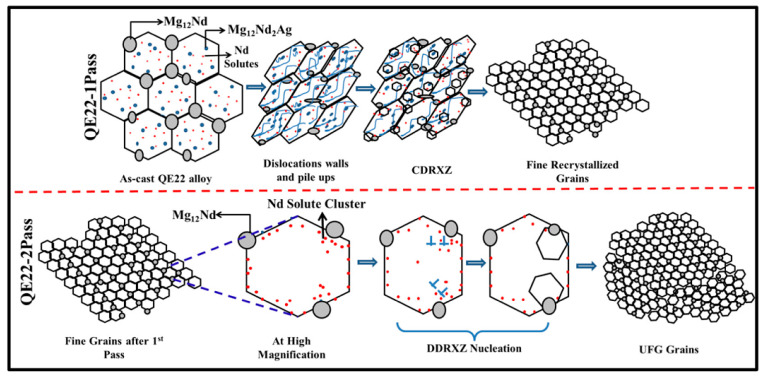
Schematic of the grain refinement process during FSP of the QE22 alloy (Reprinted with permission from ref. [99]. Copyright 2018 Elsevier Inc.).

Similarly, Khan et al. [99] employed multi-pass FSP under a quick cooling system to prepare a cast QE22 Mg alloy with the UFG microstructure. In three steps, they detailed the development of UFG structures in the QE22 Mg alloy. (i) Fine recrystallized grains, homogeneous distribution of fine Mg_12_Nd eutectics, and dislocation walls are injected into the SZ by continuous DRXZ during QE22-1Pass. (ii) Due to the obvious high strain rate and pre-existing microstructural defects and peculiarities, abundant nucleus production occurs via discontinuous DRXZ after QE22-2Pass. (iii) Due to the obvious significant solute drag and Zener pinning impact on the grain boundaries, as well as the quick removal of heat via an auxiliary cooling system, the recrystallized grains only develop partially (copper plate and compressed air). Throughout QE22-1Pass and QE22-2Pass, the procedures for an evolution of the UFG via the recrystallization mechanism are depicted in Figure 4 [99].

**Figure 5:**
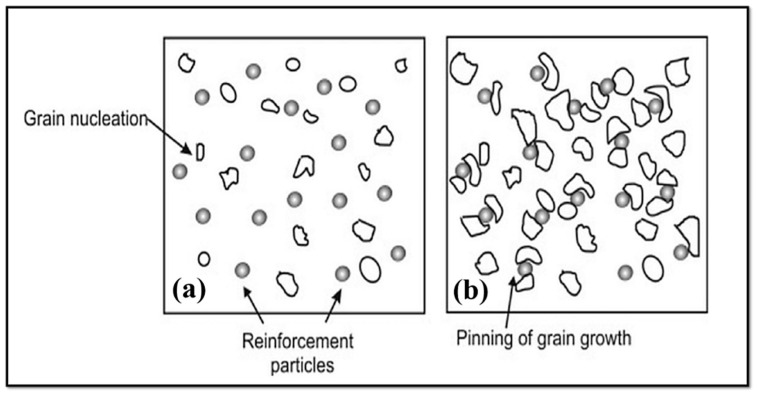
Retardation mechanism in grain growth during composite manufacturing in FSW/FSP technique: (**a**) grains nucleation and (**b**) pinning of grain boundaries due to reinforcements (Reprinted with permission from ref. [95]. Copyright 2015 Elsevier B.V.).

Manufacturing composite via the addition of nano/micro-particles results in remarkable grain refinement in the stir zone. Since these particles hinder grain growth by pinning grain boundaries [95,96], this mechanism is shown in Figure 5 [95]. In Figure 5a, grains nucleate, and in Figure 5b, grain growth is stopped via the pinning effect of reinforcement particles [95]. Equation (1) expresses that the limitation of grain size (*R*) by pinning of grain boundaries is directly proportional to the mean radius of added particles (*r*) and is inversely proportional to the volume fraction of particles added (i.e., *f*) [95,96]:

(1) R=4r/3f

Therefore, a decrease in the mean size of added particles and an increase in their volume fraction can drastically improve grain refinement. It was reported [96] that nanoparticles could significantly prevent unavoidable welding defects. Composite manufacturing via FSW/FSP treatment improves properties of the material and also joint properties resulting from FSW [96]. Eivani et al. [100] used FSP to create a WE43 Mg alloy encapsulated with hardystonite particles, and their results showed that the WE43 alloy’s compressive strength and strain-at-failure escalated due to the combination of the FSP and hardystonite fillers. Similarly, Qin et al. [101] employed FSP with a rotational speed of 6000 rpm to refine the structure and encapsulate nano-hydroxyapatite (nHA) fillers to change surface characteristics of Mg alloy. Hardness testing revealed that the dispersion nHA had a remarkable impact on the surface layer’s hardness, where the hardness value depends on the nHA content. Eivani et al. [102] point out that using FSP resulted in a considerable reduction in grain size. After two runs of FSP, the grain size was diminished from 12.4 mm to 2.5 mm. The use of two runs of FSP resulted in significant second-phase particle fragmentation. FSP could also redistribute these particles successfully. In comparable work conducted by Dinaharan et al. [103], FSP was also utilized to prepare AZ31-based composite containing Ti-6Al-4V particles reinforced. Due to dynamic recrystallization and the pinning impact of smaller size and fragmented Ti-6Al-4V particles, the grains in the composites showed exceptional refinement, which led to augmentation in UTS from 226 MPa (bare sample) to 322 MPa for a sample containing 21 vol.% Ti-6Al-4V particles. Table 2 confirmed that the addition of reinforcement to the material during FSW reduces the mean grain size. Moreover, a summary of nanoparticles reinforced materials subjected to the FSW and FSP is presented in Table 3.

Generally, no fusion and no solidification will happen in FSW/FSP [2,27,28,96]. For this reason, FSW and FSP are the best solid-state friction stir techniques [27,112]. Moreover, the existing defects associated with the fusion and the solidification of fusion welding processes such as cracks, shrinkages cavities, porosities, lack of fusion, lack of penetration, formation of detrimental phases, and so on will not appear in FSW/FSP [2,27,28,96]. Table 4 summarizes other advantages of the FSW/FSP technique.

## 4. Parametric Influence concerning FSW/FSP of Mg-Based Alloys

The parameters of FSW/FSP play an important role in the final properties of the processed or welded alloy, and they should be chosen optimally to be attained the best properties [2,27,28,32,34,72]. The most prevalent parameters are mainly: rotation speed, traveling speed, tilt angle, tool offset, axial downward force, tool profiles, pin length, pin diameter, shoulder diameter, shoulder diameter to the pin diameter ratio, shoulder morphology, shoulder angle, pin morphology, pin angle, rotation direction, cooling media, the geometry of shoulder and pin, sheet thickness, and chemical composition [2,27,28,32,34,72]. Species of geometry and morphology of shoulder and pin can be seen in Figure 1e. On the other hand, when the FSW/FSP process makes a composite structure, other variables are added besides the abovementioned parameters. These additional variables include reinforcement particles size, particles volume fraction, particle morphology, particle chemical composition, and the number of passes [27,95,96]. The rotating speed controls the action of stirring, frictional heat, plastic deformation, and intermixing of the softened material. The transverse speed influences the behavior of material flow, heat, and joining phenomenon. The tilt angle and the tool offset also affect the formation of a bond, forging, or action of stirring and optimal distribution of heat and stresses during FSW of dissimilar alloys. On the other hand, the downward force maintains the surfaces between tool and material in contact with each other and shares generated heat caused by friction. The shoulder morphology plays a role in the mixing and stirring of metal at the joining surface. A shoulder with a complex morphology can contribute to better flow of the material. The shoulder diameter is responsible for heat generation, material transfer, and plastic deformation. More heat is produced by increasing the shoulder diameter. The shoulder angle is effective on the material entrapment. The concave angle can accumulate material better, and an increase in concave angle leads to an overflow in the material. The mixing and transferring of materials around the pin are affected by the pin morphology. Like the shoulder, the complex morphology of the pin leads to more heat generation and easier flow of the material. The pin angle also controls the transfer of materials along with the thickness of the sample. An increase in the pin angle increases the flow of the material. Like the shoulder diameter, the pin diameter also plays a significant role in heat production and plastic deformation. Increasing the pin diameter causes an increase in heat generation but decreases the flow of the material. The pin length has a great effect on the forging and stirring of the material. The effects of forging and stirring of the material become more severe by an increase in the pin length, and defects are prevented more effectively. On the other hand, the generated heat increases with increasing the hardness of the tool material, as well [27,28,32]. It should be described that grain size evolution during FSW/FSP is a function of material properties, environmental conditions, and process parameters such as rotation speed and transverse speed [1,27]. Moreover, it is necessary to state that temperature, deformation rate, and cooling rate are dependent on process parameters during these techniques [27,28,45]. A brief summary of the work carried out regarding parameters that affect FSW and FSP techniques for Mg-based alloys is summarized in Table 5. In the end, the peak temperature (i.e., *T*) in the stir zone can be estimated using some parameters in the form of Equation (2) [3]:

(2) $$T=\;K{\left(\omega^{2}/v\cdot 10^{4}\right)}^{\alpha }\cdot T_{m}$$

where $T_{m}$, v, ω, K, and α are the melting point of base metal, the processing/welding speed, the rotation speed, a constant in the range of 0.65–0.75, and a constant in the range of 0.04–0.06, respectively. Moreover, heat input, i.e., *HI* (in KJ/mm) in FSW/FSP can be calculated by Equation (3) [32]:

(3) $$HI=Q_{Total}/v=Q_{Shoulder}/0.83v=2\pi \mu F_{n}R_{i}\omega /0.83v$$

where v, $Q_{Total}$, $Q_{Shoulder}$, μ, $F_{n}$, $R_{i}$, and ω are the welding/processing speed (in mm/min), total heat produced, the heat produced by tool shoulder, friction coefficient (usually 0.3), axial downward force (in kN), the radius of tool shoulder (in m), and the rotation speed (in rpm), respectively.

## 5. Applications

**Figure 6:**
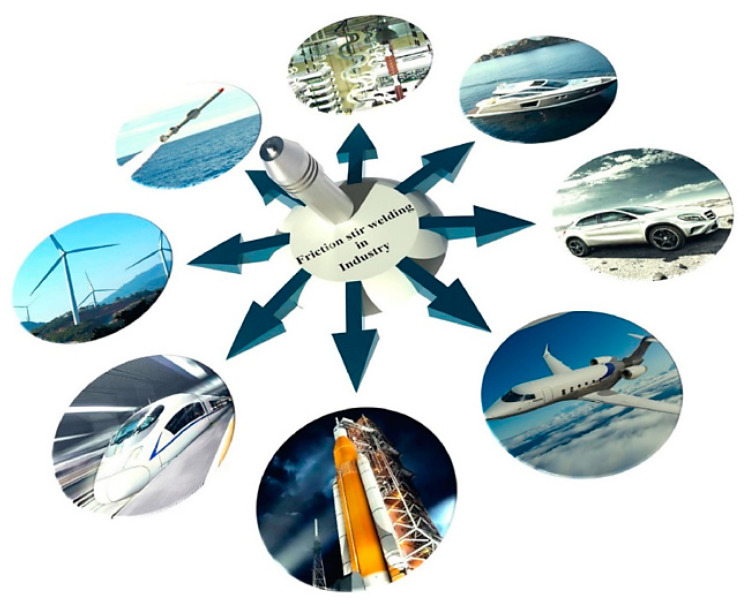
Applications of FSW/FSP technique in railway, aerospace, automotive, renewable energy, shipbuilding and marine, and defense industries (Reprinted with permission from ref. [28]. Copyright 2020 Elsevier Ltd.).

As discussed earlier, Mg-based alloys and FSW and FSP methods are used in various industries. Because Mg-based alloys have a high strength-to-weight ratio, they are extensively used in the automobile industry [119]. Thus, the fuel consumption of the vehicle is reduced using them [1,2,3,4,6,120]. On the other hand, these alloys possess suitable biocompatibility and are applicable as a biomaterial. In other words, it is a bioabsorbable material such as those for stents and bone implants [9,10,11,12,13,14,121]. The applications of FSW/FSP technique in various fields could be observed in Figure 6 [28]. Other principal utilization of Mg alloys, as well as the FSW/FSP technique could be illustrated as follows [2]:

(a) Construction industries such as pipelines, bridges, reactors for power plants, and frames.
(b) Railway industries such as container bodies, trams, wagons, and underground carriages.
(c) Land transportations such as track bodies, engine chassis, tail lifts for tracks, wheel rims, body frames, fuel tankers, and mobile cranes.
(d) Shipbuilding and marine industries such as marine and transport structures, panels for decks and floors, masts and booms for sailing boats, helicopter platforms, hulls and superstructures, and offshore accommodation.
(e) Aerospace industries such as cryogenic tanks for space vehicles, wings, aviation fuel tanks, fuselages, and fuel tanks.
(f) Other Industries such as motor housing.

## 6. Microstructural Assessment

**Figure 7:**
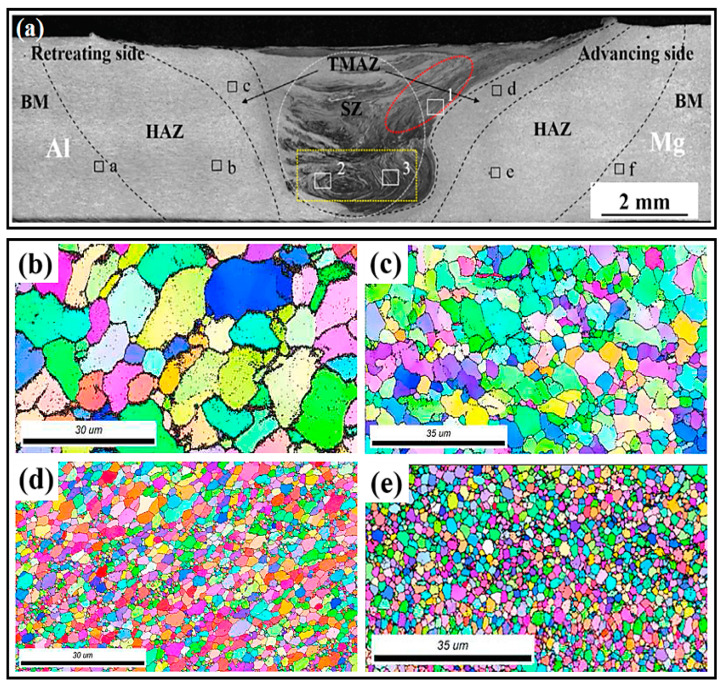
(**a**) Optical macrograph of the transverse cross-section of the Al-Mg joint welded in air with FSW (Reprinted with permission from ref. [14]. Copyright 2020 Elsevier B.V.), and EBSD maps of the grain structures of the FSP stir zone magnesium alloy samples after (**b**) 1 pass, (**c**) 2 passes, (**d**) 4 passes and (**e**) 6 passes of FSP (Reprinted with permission from ref. [102]. Copyright 2015 Elsevier Ltd.).

Microstructural studies after FSW/FSP show that four different zones due to the various thermal cycles and deformation can be identified: (1) base metal (BM), (2) heat-affected zone (HAZ), (3) thermomechanically affected zone (TMAZ), and (4) stir zone (SZ) or nugget zone (NZ) [2,27,28]. These created zones have different characteristics such as grain size; dislocations density; residual stress; and precipitations size, shape, and distribution [2,27,28]. These zones are presented in the figure where BM does not experience any heat and deformation as shown in Figure 7a [14]. HAZ is positioned between BM and TMAZ where, in HAZ, the material undergoes the effects of thermal cycles, and there is no mechanical stirring and plastic deformation in this zone. It is worth noting that grains become coarsen and metallurgical transformations occurred in this zone. In FSW/FSP, HAZ is very narrow compared to other fusion welding processes which are caused by restricted and very low heat input in FSW/FSP. TMAZ is a narrow area around SZ where in this zone, the temperature is high and plastic deformation occurs. Therefore, the grains are plastically deformed at high temperatures and are elongated in this zone. This is caused by the interaction between the rotary tool and material. It has been stated that TMAZ is a heavily elongated structure in the upward flow pattern in surrounding SZ. Reduction of the distance of this zone from SZ causes an increase in the deformation of grains. It is vital to say that this amount of plastic deformation is small, and recrystallization does not usually occur due to inadequate strain in TMAZ. The grain size in this zone is larger compared to SZ. On the other hand, there was confirmed a grain size gradient in TMAZ, which is likely related to inadequate deformation and heat exposure. SZ is the result of severe material flow or stirring that is due to tool rotation. Because in this zone, the material tolerates intense thermomechanical treatment, therefore, the recrystallization and recovery processes are occurred. It was reported [2,27,28] that a fine-grained or ultrafine-grained structure is developed with the occurrence of DRX in this zone. It has been shown that there are a large number of sub-grains and a high density of sub-grain boundaries and dislocations in stir zone (SZ). Moreover, the SZ/BM interface on the advancing side (AS) and the retreating side (RS) is wholly sharp and fairly diffusional, respectively [2,27,28]. Wang et al. [122] exhibited in TMAZ, grains have been elongated due to plastic deformation, and recrystallization has not occurred or has not been completed while, in the SZ, equiaxed and fine grains are observed. Microstructural studies also identified the upper shoulder affected zone (USAZ) and lowered shoulder affected zone (LSAZ) besides the aforementioned zones. The grain size in these two zones is larger than that of the SZ center. The cause can be attributed to the more heat produced close to the shoulder affected zone (SAZ) compared to the center of SZ [28]. Figure 7b–e depicts EBSD micrographs of the grain structure in the stir zone (SZ) of magnesium alloy specimens after 1, 2, 4, and 6 passes of FSP [102]. Throughout the FSP, there was a gradual decline in grain size from 5.47 to 1.41 μm. When compared to the grain size of the as-annealed specimen (12.4 μm), the first and second pass of FSP resulted in substantial lessening in grain size. Grain refinement progressed to achieve a minimum of 1.4 μm with additional deformation, which displayed an 85% lessening from the as-annealed grain size. The alloy's ductility and strength could be significantly enhanced with such a considerable lessening in grain size. However, there are less significant differences in grain size after 4, and 6 passes of FSP, implying that grain refinement had reached its limit. Table 6 also proves that FSW and FSP can transform the structure of coarse-grained BM to a fine-grained and ultrafine-grained structure in SZ.

It should be mentioned that approximately 80% of the work arising from the plastic flow is squandered as the heating gives rise to the local adiabatic heating [27]. It is worth noting that the thermal field, strain, and strain rate applied are considerably heterogeneous. These cases depend on and alter with distance from the pin and shoulder concerning the tool geometry, rotation speed, the transverse speed, the axial force of shoulder on the workpiece, the tilt angle, and the properties of the workpiece such as thermal conductivity, the temperature dependence of the flow stress, etc. [27]. The highest peak temperature, strain, and strain rate belong to the surfaces close to the pin and shoulder and remarkably decrease toward BM. The temperature peak at SZ decreases from 0.9 to 0.75 Tm (melting point) away from the contact surface of the shoulder and away from the surface of the pin in the transverse direction. SZ displays a pseudo-basin shape that is strikingly widened toward the upper surface. SZ is not symmetrical relative to the welding line, as well. The temperature peak in TMAZ is reduced from −0.7 to 0.6 Tm with distance from the welding line [27]. It is known that TMAZ is a transition zone where the temperature drastically changes across it. Moreover, the peak temperature in HAZ is progressively reduced from −0.55 Tm to the room temperature from TMAZ to BM. It can be declared that the gradients of the strain and strain rate are much sharper than the gradients of temperature [27]. The true strain and strain rate in SZ could be as large as $\geq 10^{2}$ and >10 $s^{-1}$, respectively, lessening downwards from the surface of the shoulder and away from the surface of the pin in the transverse direction. Strain and strain rate in TMAZ is also reduced towards zero in the boundary with HAZ [27]. However, it can be stated that here strain is large enough to cause a tangible deformation of the microstructure [27].

**Figure 8:**
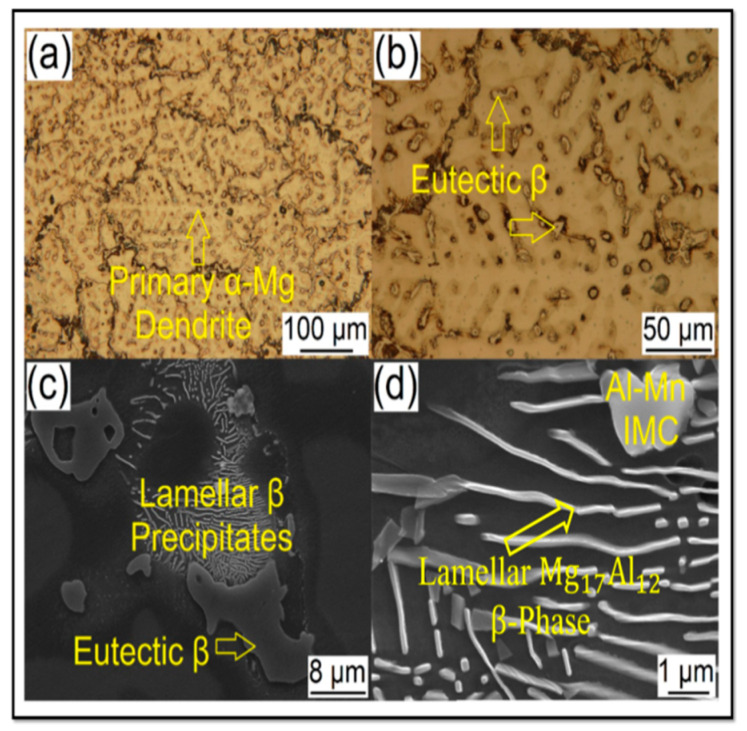
Microstructure of the as-cast AZ91 alloy: (**a**,**b**) optical microscopy (OM) images, and (**c**,**d**) scanning electron microscopy (SEM) images (Reprinted with permission from ref. [15]. Copyright 2020 Elsevier B.V.).

**Figure 9:**
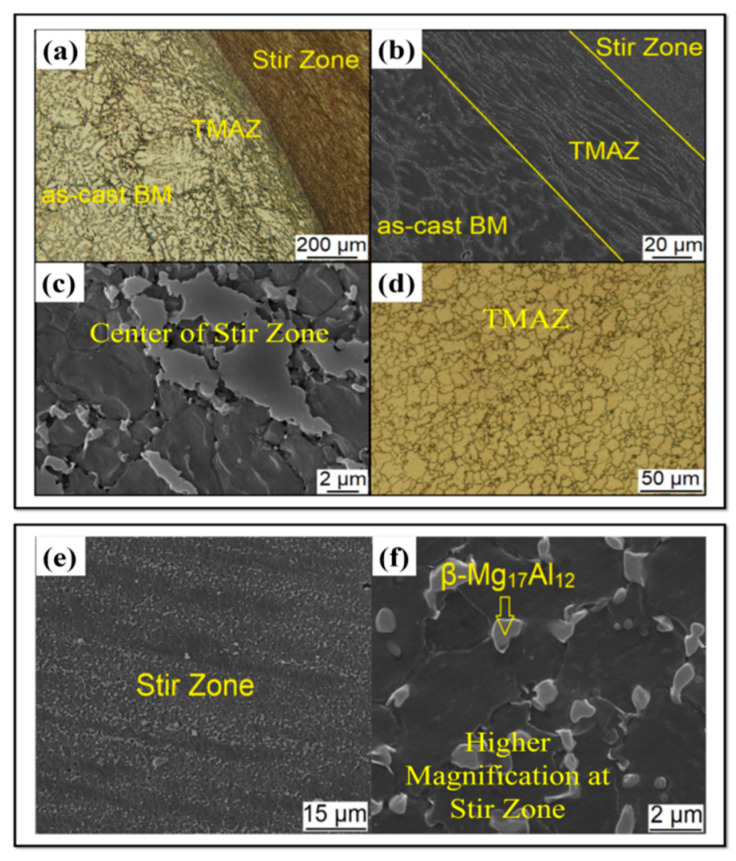
Microstructure of AZ91 alloy friction stir processed: (**a**) OM image of various zones, (**b**) SEM image of various zones, (**c**) SEM image of center of SZ, (**d**) OM image of TMAZ, and (**e**,**f**) SEM images of SZ at higher magnification (Reprinted with permission from ref. [15]. Copyright 2020 Elsevier B.V.).

Yousefpour et al. [130] showed the average grain size substantially diminished from 61.6 μm for the as-cast sample to less than 10 μm for the FSPed AZ91 alloy as a result of the incident of DRX. The FSP was also employed by Zhang et al. [131] to refine the microstructure of AZ91 alloy. Due to the incident of DRX and the plate-like beta phase fracture into small particles, the α-Mg grains are extremely refined to fine equiaxed grains with a grain size of around 3 μm [53]. In a similar work, FSP of cast alloy AZ61 [132] and ZKX50 [133] showed considerable structure refinement. The texture-dependent tensile characteristics of the SZ in the FSPed AZ31B Mg alloy were also reported by Woo et al. [134]. In this respect, FSP was employed by Rokkala et al. [135] to modify the surface features of Mg-1Zn-2Dy alloy as a biodegradable implant. Due to the DRX during FSP, the grain size of the stir zone was significantly reduced, and the FSPed alloy presented greater wettability compared to the as-cast alloy. Wang et al. [136] performed the FSP on cast Mg-6Zn-1Y0.5Zr alloy. The coarse eutectic I-phase network was dissolving, and grain refinement was reported in their study, which enhanced the tensile characteristic of the FSPed Mg alloy. The influence of FSP on the microstructure and mechanical characteristics of the as-cast Mg-Al-RE (AE42) alloy was studied by Jin et al. [137]. They reported enhancement in mechanical performance as a result of FSP. Luo et al. [132] found that due to the incident of DRX, the grain size of the as-cast AZ61 plate was greatly refined using FSP. The grain size of stir zones may be refined further in subsequent treatment, whereas periodic transition zones with heterogeneous microstructures were detected in the plate. To produce fine-grained AE42 Mg alloy, Arora et al. [138] implemented forced cooling FSP. Their study presented that the second phase particles were refined to 50 nm, which produced the pinning impact and amplified the microhardness. Dobriyal et al. [139] reported grain size refinement and precipitates in the FSWed AE42 Mg alloy. FSPed Mg alloys incorporated with rare earth (RE) exhibit substantially higher elongations compared to the FSPed Mg alloys without RE. This is because the grain structures of FSPed Mg alloys incorporated with RE containing fine precipitates are more stable throughout superplastic deformation in comparison with FSPed Mg alloys without RE [18,99,140]. Microstructural studies indicate that various and considerable microstructural changes take place by FSW/FSP in material [2,15,27,28,32]. Figure 8 shows the microstructure of an as-cast AZ91 alloy [15]. It can be observed that the microstructure of the alloy consists of a solid solution phase of α-Mg (dendrites) that the secondary phase, i.e., ꞵ-eutectic with the composition of ${\mathrm{Mg}}_{17}{\mathrm{Al}}_{12}$ has been distributed between interdendritic zones [15]. This ꞵ-eutectic is coarse (grain size is 174 ± 9 μm), and also, ${\mathrm{Mg}}_{17}{\mathrm{Al}}_{12}$ precipitations have been formed as pseudo-pearlite (layered-shaped precipitations) in the microstructure. On the other hand, a small fraction of the intermetallic compound of Al-Mn was also formed. Then, this alloy was subjected to FSP [15]. As can be seen in Figure 9, SZ, TMAZ, and BM are separated from each other [15]. The SZ microstructure indicates three significant characteristics as compared to the BM microstructure, as shown in Figure 8 and Figure 9e–f [15]: (1) The dendritic structure has been removed, and grain refinement has been obtained due to the occurrence of DRX so that the grain size decreases from the as-cast state to the friction stir processed state, i.e., from 174 ± 9 μm to 5 ± 0.4 μm, respectively [15]. (2) The ꞵ phase of ${\mathrm{Mg}}_{17}{\mathrm{Al}}_{12}$ has been fragmented and has been transformed into spherical and ultrafine particles with a size of 0.2 ± 0.67 μm. The reason for it is SPD caused by FSP. These ultrafine particles can hinder the growth of the grain of the matrix phase during the cooling step. In this way, ultrafine particles of the ꞵ phase contribute to the grain refinement in SZ [15]. (3) The ꞵ-eutectic phase of ${\mathrm{Mg}}_{17}{\mathrm{Al}}_{12}$ is dissolved in the matrix because the volume fraction of this phase has decreased after FSP so that SZ does not contain the ꞵ phase at all, indicating the ꞵ phase has been dissolved [15]. It should be said that the ꞵ phase precipitations and ꞵ-eutectic possess different dissolution behavior due to different thermal stability, in other words, different dissolution temperatures [15]. Based on Figure 9, the structure has been plastically deformed, but DRX has not happened in TMAZ. Therefore, it would be expressed that significant microstructural evolutions occur during FSW/FSP, including grain refinement, plastic deformation, shear and fragmentation of phases and precipitations, dissolution of phases and precipitations in the matrix phase, and so on. These phenomena absolutely affect the properties of the friction stir processed/welded material [15].

**Figure 10:**
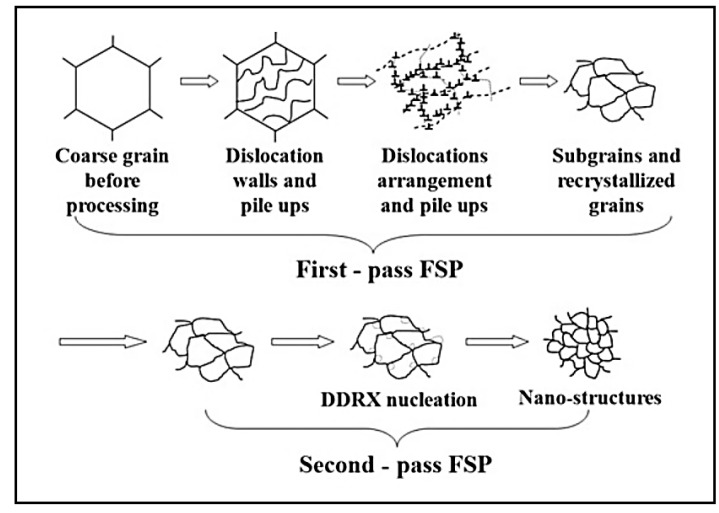
Schematic illustration of the grain refinement process of the two-pass FSP AZ31 Mg specimens (Reprinted with permission from ref. [54]. Copyright 2008 Acta Materialia Inc.).

Investigations revealed that the nanostructures could be reached via doing two passes of FSP [27,54]. Its mechanism is shown in Figure 10 [27,54]. Firstly, by performing the first pass, high strain zones are created with dislocations walls, sub-grains, as well as grains resulting from continuous DRX (CDRX). Then, the second pass is caused by lower heat input, so high total strain and strain rate lead to the creation of additional nucleation locations by discontinuous DRX (DDRX) mechanism. Accordingly, meaningful grain refinement is made, and it can be reached to a nanostructure, as can be seen in Figure 10 [27,54].

**Figure 11:**
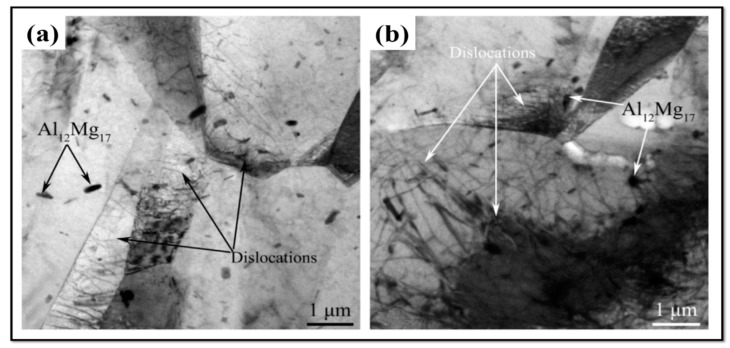
TEM images of dislocations distribution at SZ of the friction stir processed AZ31 alloy: (**a**) rotation speed of 1000 rpm—processing speed of 25 mm/min and (**b**) rotation speed of 5000 rpm—processing speed of 125 mm/min (Reprinted with permission from ref. [3]. Copyright 2020 Elsevier B.V.).

**Figure 12:**
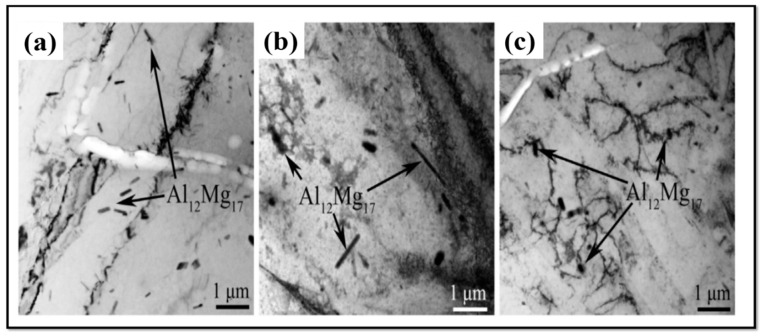
TEM images of precipitations distribution at SZ of AZ31 alloy friction stir processed: (**a**) rotation speed of 1000 rpm—processing speed of 25 mm/min, (**b**) rotation speed of 5000 rpm—processing speed of 125 mm/min, and (**c**) AZ31 alloy unprocessed (Reprinted with permission from ref. [3]. Copyright 2020 Elsevier B.V.).

**Figure 13:**
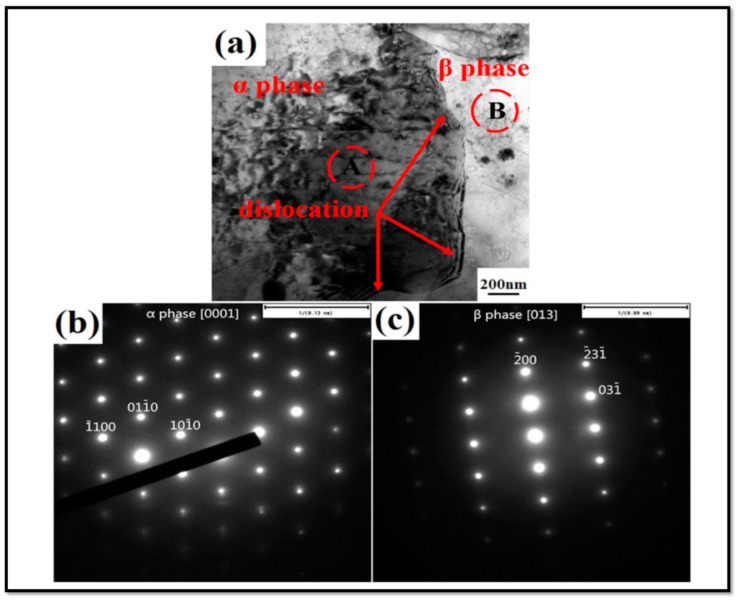
TEM image and SAED patterns at SZ of the friction stir processed LZ91 alloy: (**a**) bright field image, (**b**) SAED pattern of A zone, and (**c**) SAED pattern of B zone (Reprinted with permission from ref. [24]. Copyright 2018 Elsevier B.V.).

Observations by transmission electron microscopy (TEM) show that a substructure of dislocations can be developed during FSW/FSP [6,27,28]. Figure 11 is a TEM image of SZ of a friction stir processed AZ31 alloy [3]. Figure 11a is related to the rotation speed of 1000 rpm and the processing speed of 25 mm/min. It is observed that there is a high fraction of sub-grains in the SZ, and the density of dislocations is low inside grains [3]. In contrast, Figure 11b corresponds to the rotation speed of 5000 rpm and the processing speed of 125 mm/min. In Figure 11b, the fraction of sub-grains is smaller, but there is a high density of dislocations inside the grains and at the grain boundaries [3]. Moreover, the grain size in this condition (i.e., Figure 11b) is larger than that of the previous condition (i.e., Figure 11a) [3]. The TEM image in Figure 12 also represents the characteristics of the distribution of ${\mathrm{Mg}}_{17}{\mathrm{Al}}_{12}$ precipitations in SZ and BM of the friction stir processed AZ31 alloy [3]. When this alloy is subjected to FSP, a high fraction of the ${\mathrm{Mg}}_{17}{\mathrm{Al}}_{12}$ precipitations are seen in SZ. Meanwhile, increasing the rotation speed and the processing speed leads to the growth of part of these precipitations [3]. It can be said that the distribution of ${\mathrm{Mg}}_{17}{\mathrm{Al}}_{12}$ precipitations is pseudo-networked [3]. TEM image and selected area electron diffraction (SAED) pattern of SZ of the friction stir processed LZ91 alloy are shown in Figure 13 [24]. The LZ91 alloy consists of two phases of α-Mg and ꞵ-Li. Concerning Figure 13a, the interphase boundary between two phases can be obviously seen. Figure 13b,c illustrates the SAED pattern of A and B zones in Figure 13a, respectively [24]. It is found, based on Figure 13b,c, that the black and white zones are associated with α-Mg phase with the HCP structure and the ꞵ-Li phase with the body centered cubic (BCC) structure, respectively. A small fraction of dislocations have been distributed close to the boundary of the α-Mg phase, as shown in Figure 13a [24]. Based on Figure 13a, it can be stated that the density of dislocations is rather high inside the α-Mg phase, while there is a small number of dislocations density inside the ꞵ-Li phase. The reason is that the ꞵ-Li phase with BCC structure has a higher softness and more plasticity compared to the α-Mg phase with HCP structure [24]. Therefore, the plastic deformation is preferably performed in the ꞵ-Li phase during FSP, and this phase gets sooner and easier to the required critical strain for the occurrence of DRX. As a result, DRX more fully takes place in it [24]. In general, it is worth noting that FSW/FSP leads to an evolution in distribution and density of dislocations as well as characteristics and distribution of phases and precipitations.

**Figure 14:**
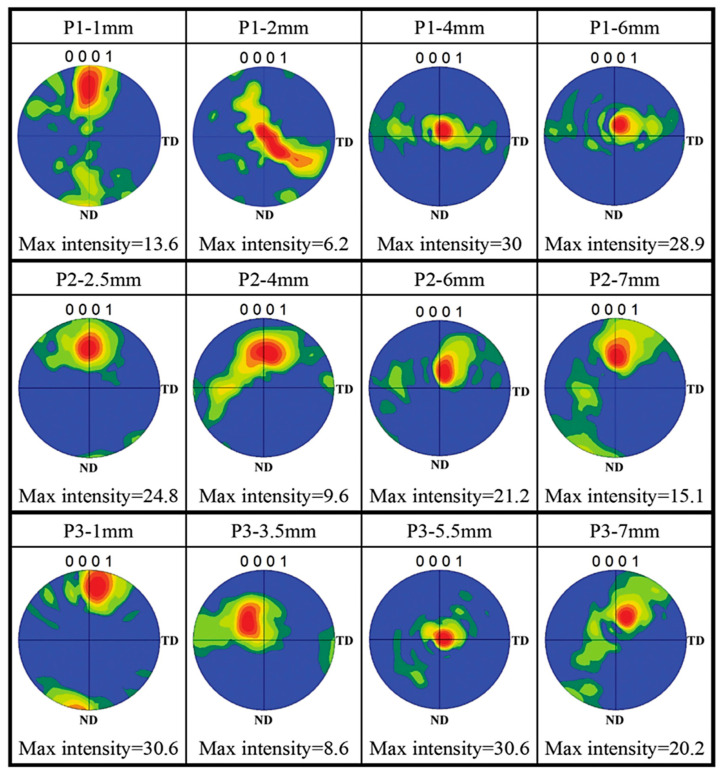
Pole figures of (0001) derived from EBSD for various locations on the cross-sections of NZ30K alloy friction stir processed in different rotation speeds and processing speeds (Reprinted with permission from ref. [19]. Copyright 2016 Elsevier Ltd.).

During FSW/FSP, SPD is accomplished in the material, the temperature rises high enough, and DRX also occurs. Therefore, it is anticipated that evolutions take place in SZ, TMAZ, and their texture [1,2,9,27,28]. It was reported that initial texture has not great impact on the final microstructure and texture of SZ, and various distributions of textures formed in the TMAZ possess an important role in the mechanical and fracture behavior of material [2]. It has been shown that in AZ31B-H24 alloy, there is a strong texture with basal planes (0002) mostly parallel to the rolling plane and $\langle 11\bar{2}0\rangle$ directions aligned in the rolling directions [2]. Then, after FSW, base planes in SZ slightly tilted toward the transverse direction (specified from the normal direction, i.e., the top surface) and also slightly tilted toward the rolling direction (specified from the transverse direction, i.e., cross-section) [2]. It can be due to severe shear plastic flow close to the surface of the pin tool [2]. On the other hand, the fiber textures are also created in the (10$\bar{1}$0) prismatic planes and (10$\bar{1}$1) pyramidal plates [2]. Commin et al. [141] stated that in the hot rolled AZ31 alloy, the normal of {0002} basal plane was parallel to the normal direction of the material. They said that after FSW with the shoulder diameter of 13 mm, significant modifications were not made, while the shoulder diameter of 10 mm led to a modification in the strong basal textures, i.e., {0002} [141] so that the base plane is gradually orientated perpendicular to the direction of welding moving toward SZ [141]. Figure 14 depicts (0001) pole figures derived from EBSD for various locations on the cross-sections of the friction stir processed NZ30K alloy in different rotation speeds and processing speeds [19]. P1, P2, and P3 belong to the rotation speed and the processing speed of 800 rpm-200 mm/min, 1000 rpm-150 mm/min, and 1200 rpm-100 mm/min, respectively. This states that the rotation speed increases, and the processing speed decreases from P1 to P3. In Figure 14, P1-1mm states that the detecting point is 1 mm away from the upper surface in the P1 group, and the same concept is established for the rest of the cases [19]. It is observed that in the upper layers and in the first column, a fairly strong texture close to –〈0001〉∥ ND is cognately created in all of the groups. It can be said that regarding the middle layers in the third column, a 〈0001〉∥ PD strong texture is established in all of the groups. This is associated with the shear deformation created by the rotating pin and the related alignment of the base planes with the columnar surface of the pin. In the lower layers and the fourth column, the texture corresponding to the middle areas remains strong while there is a small rotation of the texture towards the ND direction. This can be likely attributed to the downwards reaction of the material unreformed [19]. Moreover, there is a substantial point between the top and middle layers in the second column, which is not anticipated. This happens where the intensity of the texture is intrusively weakened as compared to its adjacent situations. Such areas are transition regions between the regions of pin driven and shoulder driven where the effect of shoulder and pin results in texture weakening/softening of 〈0001〉∥ ND and texture strengthening of 〈0001〉∥ PD, respectively. In general, the intensity of the texture obtained in the transition region is comparatively lower than that of other regions [19]. According to Figure 14, it can be found that the texture changes in sites around 3–4 mm away from the upper surface between P1 and P2/P3. P1-4 mm displays 〈0001〉∥ PD strong texture that is like the texture of P2-6 mm and P3-5.5 mm. This simply expresses that the transition regions progress greater from P1 to P2/P3. This can probably be attributed to the material softening caused by comparatively further heat input during FSP [19]. Liu et al. [3] investigated the FSP behavior of AZ31 alloy under the same speed ratio. The results showed that the average grain size and the fraction of $\beta -{\mathrm{Al}}_{12}{\mathrm{Mg}}_{17}$ precipitations were smaller and higher as compared to BM, respectively [3]. Although grains and precipitations coarsen by increasing the processing speed, the distribution and homogeneity of precipitations improve due to proper and adequate heat input. On the other hand, DRX results in modifying and randomizing texture in SZ [3]. Gotawala et al. [1] investigated the effect of multi-pass FSP with the spiral strategy on Mg-3Al-0.2Ce alloy. They showed that there were two discrete areas in the cross-section of the processed blank [1]: banded and non-banded areas. The grain size in banded areas is much smaller than that of non-banded areas. The banded areas become more evident by increasing the rotation speed of the tool and overlap tool [1]. Moreover, by an increase in the overlap tool speed, the difference in the grain size is highlighted in the mentioned areas and identification of interface between them is facilitated. This is related to the bimodal microstructure [1]. Generally, it was concluded that multi-pass FSP causes a formation of bimodal microstructure and strong texture in comparison with single-pass FSP [1]. The cause of texture creation is an increment of the texture index caused by the close packing of a similar microstructure resulting from the retreating side of successive passes [1]. Finally, it is concluded that FSW/FSP can lead to a remarkable evolution in the type and intensity of the texture. Moreover, the phenomena of texture softening and texture strengthening may take place depending on the FSW/FSP variables and alloy conditions [20,142,143].

## 7. Mechanical Performance

As discussed in the previous section, FSW/FSP causes widespread changes in the microstructure, substructure, and texture of the material. It was mentioned that even by FSP (or FSW), the surface of the material could be transformed into a composite. Therefore, it is expected that the mechanical properties of the material friction stir welded/processed vary dramatically [18,26,27,34,44,109,118,144]. When any alloy like Mg alloys are subjected to the FSW/FSP, some strengthening mechanisms are developed in them by these techniques [2,15,27,28,38]. These strengthening mechanisms are as follows [15]:

(1) Grain refinement strengthening: also known as grain boundary strengthening and Hall–Petch strengthening. It was expressed that a fine-grained or ultra-fine-grained structure is formed in SZ. Therefore, the boundary pinning coefficient, i.e., Hall–Petch coefficient, is very high. As a result, the strength improves [15].

(2) Orowan strengthening: this mechanism is activated when secondary particles are distributed in the microstructure by FSW/FSP. These particles are precipitations that were formed before FSW/FSP, either induced into the material by composite making or formed during FSW/FSP. In this case, dislocation-particle reactions (i.e., Orowan mechanism) are activated. It should be noted that precipitations or phases which have been already formed by FSW/FSP can be fine or ultrafine. This increases the intensity of dislocation-particle reactions and results in pinning of grain boundaries—consequently, the strength increases [15,145].

(3) Solid solution strengthening: It is reported that precipitations and secondary phases might be dissolved inside the matrix phase during FSW/FSP, and there are also the effects of homogenization associated with these techniques. Thus, the concentration of some alloying elements increases within the matrix phase. This leads to an increment in strength [15,145].

The yield strength of the alloy friction stir welded/processed can be considered as a summation of several terms, which are summarized in the form of Equation (4) [15]:

(4) $$\sigma_{y}=\Delta \sigma_{CRSS}+\Delta \sigma_{Orowan}+\Delta \sigma_{SS}+\Delta \sigma_{GB}$$

where $\Delta \sigma_{CRSS}$, $\Delta \sigma_{Orowan}$, $\Delta \sigma_{SS}$, and $\Delta \sigma_{GB}$ are the contributions of strengthening arising from the lattice friction, i.e., critical resolved shear stress (CRSS), Orowan mechanism, solid solution, and grain boundary (i.e., grain refinement), respectively. These mentioned terms could be estimated as follows [15]:

$\Delta \sigma_{CRSS}$: This is determined concerning the CRSS and Taylor orientation factor of the alloy [15].

$\Delta \sigma_{Orowan}$: This is Orowan-induced strengthening which is caused by interaction of the basal dislocations (in Mg alloys) with secondary phase particles. This can be estimated by the Equation (5) [15]:

(5) $$\Delta \sigma_{Orowan}=\frac{Gb}{2\pi \sqrt{1-\vartheta }\left(\frac{0.953}{\sqrt{f}}-1\right)d_{P}}\ln\left(\frac{d_{P}}{b}\right)$$

where *G*, *b*, ϑ, f, and $d_{P}$ are the shear modulus of the matrix phase (i.e., Mg alloy), the Burgers vector magnitude belonging to the gliding dislocations, the Poisson’s ration belonging to the matrix phase (i.e., Mg alloy), the volume fraction of secondary phase particles, and the average planar diameter of secondary phase particles on slip plane, respectively.

$\Delta \sigma_{SS}$: This corresponds to solid solution strengthening due to the dissolution of precipitations and secondary phases within the matrix phase (followed by increasing content of alloying elements inside it) and would be calculated by Equation (6) [15]:

(6) $$\Delta \sigma_{SS}=\underset{i}{\sum }\left(K_{i}C_{i}^{2/3}\right)$$

where $K_{i}$ and $C_{i}$ are coefficient of strengthening of component *i* that reveals strengthening potency of alloying elements and an atomic fraction of solute corresponding to the component *i*, respectively.

$\Delta \sigma_{GB}$: This is grain boundary/Hall–Petch strengthening due to grain refinement that can also be estimated by Equation (7) [15]:

(7) $$\Delta \sigma_{GB}=K_{GB}D^{-1/2}$$

where $K_{GB}$ and *D* are the Hall–Petch coefficient and the mean grain size related to SZ, respectively. Generally, it should be mentioned that these strengthening mechanisms aforementioned not only influence the yield strength but also influence the tensile strength, ductility, and fracture energy [145]. It was demonstrated [2] that by increasing the tool rotation and decreasing the welding speed, the joint strength is improved. It is also proven that among the tool materials of stainless steel, high-speed steel, armor steel, mild steel, and high carbon steel, excellent tensile properties were achieved in the tool with high carbon steel (profile of threaded pin with the shoulder diameter of 18 mm) [2]. On the other hand, it was reported that in the FSW of AZ31B-H24 alloy, strength and ductility were decreased in all strain rates [2]. Their result showed that increasing the strain rate caused a slight increase in yield and tensile strength but caused a significant decrease in the BM ductility [2]. It was reported in research on the FSW of AZ31B-H24 alloy that the hardness was reduced at the SZ center across TMAZ and HAZ [2]. The hardness reduction was caused by coarser grain size at a higher rotation speed. Moreover, the lowest hardness was in SZ, which is related to the occurrence of DRX and grain growth [2]. Their finding revealed that during FSW of Mg-Al-Ca and Mg-Zn-Y-Zr alloys, the intermetallic compounds of Al_2_Ca and phases of Mg-Zn-Y are fragmented and distributed in SZ [2]. This noticeably increases the hardness of SZ compared to other zones [2]. It should be noted that the hardness of precipitation-hardenable alloys such as ZK60 is strongly affected by precipitations (MgZn_2_) [2]. Regarding the same alloy, it is reported that the hardness of SZ was appreciably lower as compared to other zones after FSW. The cause is referred to as the further dissolution of MgAl_2_ precipitations [2]. Generally, it is reported [2] that in these cases, precipitations have a greater contribution to the hardness control of alloy than grain size. Singarapu et al. [146] stated that increasing the rotation speed initially causes an increase in the hardness and then causes a gradual decrease in AZ31B friction stir welded. They described that the hardness increment of SZ is referred to in two cases [146]: (1) the existence of a fine-grained structure and finer grains in this zone compared to other zones and (2) the precipitating of fine intermetallic compounds [146]. Sunil et al. [79] understood in FSW of AZ31 and AZ91 alloys that the hardness is gradually increased from AZ31 BM towards AZ91 BM. Moreover, many changes were made in SZ hardness which could be related to the combination of fine-grained structure and hard phase of Mg_17_Al_12_ in some zones of AZ31 alloy [79]. In general, they revealed that the hardness of SZ increased due to grain refinement, Mg_17_Al_12_ precipitations formation, and creation of the supersaturated solid solution via the more dissolution of Al element from the Mg_17_Al_12_ phase [79]. Kouadri-Henni et al. [147] showed that there are widespread changes in the transition zones of BM/HAZ, HAZ/TMAZ, and TMAZ/SZ. Thus, the hardness of HAZ close to TMAZ is similar to the hardness of BM due to the formation of the precipitation [147]. Moreover, the hardness of SZ and TMAZ is lower than that of BM, although the grain size of SZ is smaller than that of BM [147]. They indicated that the weld hardness is reduced due to effects related to the dislocations density, residual stresses, and texture [147]. It can be briefly stated about the fracture mechanisms of Mg alloys friction stir welded/processed that the fracture usually triggers in SZ or transition zone at SZ/BM interface [46]. The fracture location is affected by the presence of strong local texture in Mg alloys friction stir processed/welded [46]. The c fracture in SZ can result from specific zones with a favorable c-axis direction for basal slip [46]. However, other studies attributed the reason for fracture to the incompatible plastic deformation in TMAZ or transition zones that arise from unexpected texture alterations in the TMAZ/SZ interface [46].

Luo et al. [132] demonstrated that grain refinement and the elimination of cast flaws resulted in an excellent improvement in both strength and ductility in the FSPed Mg plate when compared to the as-cast AZ61 plate. On the AZ61 alloy, Du et al. [148] used FSP coupled with a fast heat sink to achieve the UFG structure with great mechanical performance. FSP significantly escalated the microhardness of the AZ61 alloy three times greater than the unmodified AZ61 alloy. FSP of Mg-7Al, Mg-4Al-3Ca, and Mg-2Al-5Ca alloys, according to Nasiri et al. [149], resulted in considerable structure refinement and enhanced mechanical characteristics. The increase of tensile characteristics of FSPed alloys was due to the replacement of intergranular constituents with a homogeneous distribution of fine particles. Tan et al. [8] in friction stir lap linear welding of AA6061 to NZ30K illustrated that the fracture location was along with the interface of the intermetallic layer (Al_3_Mg_2_) of Al-Mg for without post heat treatment [8]. However, they showed that this intermetallic layer was thickened after post heat treatment for 1 h, and the fracture location was also in this region [8].

At the end of this section, some case studies are described for better realization of evolution in the behavior of Mg alloys friction stir welded/processed; for example, Liu et al. [24] evaluated the microstructure and tensile properties of the Mg-9Li-1Zn alloy after FSP. It was found that the preferred direction in the α-Mg phase was changed from (0002) to (101), and DRX of β-Li phases is relatively adequate relative to α-Mg phase for BM rolled. Moreover, the grain size of BM is larger than that of SZ, and SZ grains were equiaxed and random. On the other hand, the results showed that FSP in the TD leads to a decrease in the yield strength and an increase in the tensile strength and ductility as compared to BM. The cause of improved tensile strength and ductility is the occurrence of grain refinement resulting from DRX during FSP. However, the reason for the reduction in the yield strength can be associated with texture weakening/softening, although grain refinement has occurred [24]. Jiryaei sharahi et al. [15] studied the effect of FSP on the mechanical behavior of Mg-Al-Zn (AZ91) alloy. They expressed that a single pass of FSP can improve the strength and energy absorption of this alloy. It was shown that the created sub-micron and ultrafine particles of Mg_17_Al_12_, the dissolution of Mg_17_Al_12_ intermetallic phases, and grain refinement are key factors that influence the strength and ductility of AZ91 alloy friction stir processed. Accordingly, these microstructural changes can increase strength, ductility, and energy absorption. The improved strength can be attributed to the grain refinement due to the occurrence of DRX, increment of solid solution hardening via the increasing amount of Al and Zn elements within the matrix phase through the dissolution of intermetallic phases of Mg_17_Al_12_, and increment of the intensity of Orowan mechanism through the fragmentation of mentioned intermetallic phases. The reason for improving energy absorption can also be an excellent combination of grain refinement and appropriate dispersion of fine phases of Mg_17_Al_12_. They concluded that the FSP changed the fracture mechanism from the characteristic of dimple and cleavage mixture to the characteristic of dimple [15]. Peng et al. [6] in the study of the effect of heterogeneous microstructure and texture on the mechanical properties of AZ31 alloy friction stir processed reported that grain refinement is observed in SZ due to SPD. In addition, abundant twins of {10$\bar{1}$2} and grains obtained by DRX were found, which is probably related to a complex stress state and heat dissipation. On the other hand, the irregular basal texture is modified, and an inclined basal texture is formed in SZ. This is caused by performing shear deformation around the stirring pin. Their result showed that SZ includes three layers; hence, the results of microhardness in SZ revealed that the upper and lower layers have higher hardness than the middle layer, which is referred to as the higher density of dislocations. Their result also showed that the occurrence of grain refinement and texture strengthening cause an improvement in the yield strength, ultimate tensile strength, and ductility of the samples prepared along with SZ. However, in the samples prepared in SZ, the yield strength is slightly lower than that in BM, which is associated with texture weakening. In contrast, the ultimate tensile strength and ductility are increased due to wide basal slip and twins of {10$\bar{1}$2}. Finally, they found that depending on the work hardening law, the curves of true stress–strain corresponding to the samples prepared in SZ have 5 stages that arise from the heterogeneous microstructure and texture so that in 2 and 3 stages, the dominant deformation mechanism was a basal slip. However, the prevailing deformation mechanism in stage 4 was {10$\bar{1}$2} twin. It is essential to note that the modified relations of Hollomon and Arrhenius were employed to be described the dominant stage of the plastic deformation via dislocations and twins, respectively [6]. Sing and Dubey [40] investigated the dissimilar joint behavior of AZ91 and AZ31 alloys with FSW. They said that there were no imperfections in the weld zone while flashes were also made, which were minimally higher. On the other hand, grains belonging to the AS (AZ91) were coarser in comparison with grains belonging to the RS (AZ31). They represented that the weld zone has the highest microhardness which is more than the microhardness of base alloys. The tensile test showed that the fracture location is at the AZ91 side (i.e., AS), close to the center of the joint line. Efficiency resulting from welding was 85.09% compared to the base AZ31 alloy. They also described that tensile strength and ductility of the weldment decreased relative to the base alloys, which refer to phenomena such as the dissolution of hardening phases and textural evolutions [40]. Xu et al. [85] investigated AZ31B alloy behavior under rapid cooling FSW. They concluded that cooling of liquid CO_2_ resulted in the formation of ultrafine grains with high dislocation density at the top area of the weld. Moreover, there were a large number of twins and secondary phase particles in these ultrafine grains. On the other hand, the twin’s appearance led to an extreme reduction in the intensity of basal texture. They also remarked that the top area’s strength and elongation were higher compared to the bottom area, and the welding efficiency equaled 93% [85]. Kondaiah et al. [150] made a composite with AZ31 alloy through FSP. They introduced fly ash particles into this alloy by FSP. Thus, these particles were homogeneously and heterogeneously distributed at the surface and under the surface, respectively. The microstructural study revealed that the structure is fine-grained in SZ. Their result also showed that the hardness of AZ31-Fly ash composite is significantly higher (30%) compared to AZ31 alloy. This is due to a fine-grained structure and distribution of fly ash particles. Moreover, SZ hardness was higher than that of AS and unprocessed alloy. This is caused by a fine-grained structure and the presence of fly ash particles. On the other hand, RS hardness was the highest which is attributed to the formation of more fly ash particles in RS. The wear behavior expressed that the friction coefficient in the sample prepared from RS is lower in comparison with the sample prepared from unprocessed alloy and SZ. This is associated with the higher hardness of RS. In this way, they concluded that the AZ31-fly ash composite possesses higher wear resistance as compared to AZ31 alloy [150]. It is evident that FSW and FSP techniques could have beneficial impacts on the mechanical behavior of Mg and other metallic alloys [133,151,152,153,154,155,156,157,158,159,160,161,162,163,164,165,166,167]. Hence, the mechanical properties of some types of magnesium-based alloys before and after FSP are listed in Table 7. The results of Table 7 confirm the improvement of mechanical properties of Mg alloys via FSP.

## 8. Conclusions and Future Outlook

This review article was argued around the FSW and FSP of Mg-based alloys, and research progress; grain refinement mechanisms; affecting parameters; evolution in microstructure, substructure, and texture; applications; and mechanical properties were addressed. It was concluded that the treatment parameters and the tool geometry strongly affect the mechanisms of FSW/FSP. On the other hand, these two techniques achieve extensive microstructural and sub-structural changes, and the material texture may also be softened or hardened. These intensely vary the properties of the alloy friction stir welded/processed. It should be mentioned that FSW is a solid-state welding technique, and FSP is a solid-state surface modification technique. Because in FSW, unlike fusion welding methods, fusion and solidification do not take place, defects and disadvantages regarding welding and casting are not observed. Moreover, because of adverse properties, for instance, the formation of coarse grains, brittle intermetallic compounds, cracks, voids, and oxide layers that Mg alloys exhibit under the fusion welding techniques, it could be said that the FSW is one of the best welding methods of these alloys. It is essential to mention that it is possible to convert the material surface into a composite structure with FSW/FSP. Since these methods are solid-state techniques, there are no defects associated with the composites resulting from casting. Other advantages of these methods were reported in this review article. However, the most significant characteristics and benefits corresponding to these two techniques can be the development of a fine-grained or ultrafine-grained structure accompanied by solid solution hardening as well as texture softening and strengthening. This can result in increased strength, ductility, and fracture energy of the alloy friction stir welded/processed. Overall, the tool geometry and material must be properly chosen in FSW and FSP techniques, and their parameters should also be optimally determined to improve the properties of the alloy friction stir welded/processed.

The tool geometry and material play a significant role in the alloy’s friction stir welded/processed behavior. Therefore, examination of their effects can be very useful. It is recommended that researchers study the effects of most of the FSW/FSP parameters to select optimal parameters more precisely. Joint designs in FSW have been broadly done in the form of butt and lap, and it is suggested to focus on other designs. It is evident that severe plastic deformation, frictional heat generated, and material flow are too complicated in these two techniques. Therefore, more and precise investigations and simulations need to be accomplished to make their understanding (as well as FSW/FSP mechanisms) easier. DRX is also the main reason for the grain refinement obtained by these techniques, and accordingly, the DRX mechanisms depending on the alloy and its conditions must be studied and described in detail. It should be said that, because notable residual stresses are produced in the material via FSW/FSP, post heat treatment implementation might be beneficial. Even performing heat treatment before applying these techniques and between their passes in multiple pass treatment possibly has appreciable effects on the final behavior of the material friction stir welded/processed. Moreover, the various reinforcements can be employed to be manufactured a composite structure in Mg-based alloys by FSW/FSP. This is connected with that FSW and FSP are good solid-state techniques, and problems and imperfections related to the composites obtained by casting and solidification will not exist in them. Ultimately, it would be uttered that the behavior of wear, fatigue, creep, corrosion, corrosion combined with mechanical factors as well as their mechanisms have not been wholly and accurately evaluated in Mg-based alloys friction stir welded/processed and their composites manufactured by these methods. These can be suitable topics for further research. 

## Figures and Tables

**Table 1:** Research summary of Mg-based alloys subjected to FSW/FSP.

Alloy	Processing	Conclusions/Remarks	Reference
AA6061 and NZ30K	FSW	The interface of the intermetallic compound layer of Mg and Al alloys as a failure location	[8]
Mg-6Zn-1Y-0.5Zr (wt%)	FSP	Enhancement of strength and ductility due to significant grain refinement, distribution of small second phase particles, and texture softening	[18]
GZ142K	FSP	Increment of yield strength by 30% owing to the refinement of grain and LPSO phase as well as formation of fine precipitations around the LPSO phase. Moreover, reduction of elongation by 39% due to development of the heterogeneous microstructure	[22]
AZ31B and AA6061	FSW	Mechanical strength, including tensile strength and hardness, escalated due to the grains refinement effect. In addition, excellent corrosion resistance at high rotation speed and lower welding speed	[34]
AZ31	FSP	Conversion of coarse bimodal microstructure into a fine grain and defect-free microstructure at the rotation speed of 1000 rpm occurred. Furthermore, development of a defect-free, but comparatively coarse bimodal microstructure at rotation speeds higher than 1000 rpm. Moreover, formation of finer grain sizes without the generation of voids or defects caused by increasing translational speed	[35]
Mg–7.12Zn–1.2Y–0.84Zr (wt%)	FSP	Obtaining a maximum superplasticity of 1110% at 450 °C and high strain rate of $1\times 10^{-2}\;s^{-1}$ due to the superior thermal stability of grain refinement structure and the great fraction of high angle grain boundaries	[63]
AZ31B-H24 and 2024-T3	FSW	The low microhardness value in the corroded area was due to the creation of the porous magnesium hydroxide film with microcracks. Furthermore, the occurrence of galvanic corrosion was caused by galvanic couples of Al-Mg	[75]
AZ61A	FSW	Maximum tensile strength of (83% of the base alloy) of the made welds at the axial force of 5 kN, rotation speed of 1200 rpm, and welding speed of 90 mm/min in comparison with other weld specimens	[76]
Mg–9.4Gd–4.1Y–1.2Zn–0.4Zr (wt%)	FSP	Considerable grain refinement and dissolution of large grain boundaries $\beta -{\mathrm{Mg}}_{3}$ RE and LPSO phases. Furthermore, substantial improvement of mechanical strength is obtained	[77]
Mg-30Zr (wt%)	FSP	Fragmentation of a high fraction of coarse Zr particles into smaller particles occurred. Moreover, a noticeable increment of refining efficiency due to a more desirable distribution of Zr particles is obtained.	[78]
AZ31 and AZ91	FSW	Elimination of the hot cracking via the selection of optimum welding parameters is observed	[79]
Mg–4Nd–2.5Y (wt%)	FSP	Refinement of α-Mg dendrites and fragmentation of coarse ${\mathrm{Mg}}_{12}$Nd phases into small particles occurred. In addition, excellent tensile properties caused by refinement and homogenous microstructure are obtained	[80]
Lab-prepared Mg and AA6061-T6	FSW	Enhancement of the joint strength via tailoring of banded structure	[81]
AZ31	FSW	High mechanical performance of the welds at the average rotation speed of 1200 rpm	[82]
AZ91 and A383	FSW	Defect-free FSW at the rotation speed of 900 rpm and welding speed of 40 mm/min	[83]
AZ31	FSW and FSP	Enhancement of mechanical properties of the weld caused by strengthening influence corresponding to twin lamellae and strain. Moreover, localization weakening during deformation occurred	[84]
AZ31B	FSW	Obtainment of ultrafine grains with high dislocation density in the top area of the weld owing to cooling of liquid CO_2_. Furthermore, the existence of a large number of twins and second-phase particles in these ultrafine grains is observed	[85]
Mg–Sn–Zn	FSP	The matrix grains substantially decreased in the SZ of FSPed Mg–6Sn–2Zn alloy by DRX, and the secondary phase was fragmented. The escalation of travel speed has a less significant influence on the texture of various SZ areas.	[86]
AZ61	FSP	Two-pass FSP leads to a considerable enhancement in elongation, while a slighter lessening in strength than that of one-pass FSPed AZ61 alloy which is attributed to the structure refinement, the texture evolution in the stir zone.	[87]
Mg-RE alloys	FSP	Occurrence of grain refinement, homogeneous dispersion of second-phases particles, and enhancement of mechanical properties	[88]

**Table 2:** Grain size evolution during FSW through addition of reinforcements.

Alloy	The Mean Grain Size of SZ (Before the Addition of Reinforcement)	Nanomaterial of Reinforcement	The Mean Grain Size of SZ (After the Addition of Reinforcement)	Reference
AZ31	10.5 μm	SiC	3.6 μm	[104]
AA6061andAZ31	13 μm	SiC	4 μm	[96,105]
AA6061	8.37 μm	Graphene	5.18 μm	[39]
AA5083-H111	6.6 μm	TiC	4.7 μm	[106]
AA5083	18.5 μm	$\mathrm{Ti}O_{2}$	8.5 μm	[107]

**Table 3:** Research summary of nanoparticles reinforced materials subjected to the FSW and FSP.

Alloy	Nanomaterial of Reinforcement	Conclusions/Remarks	Reference
AZ31B	SiC	Increment of tensile strength and negative effect of excessive agglomeration of nanoparticles on tensile strength	[33]
AZ31 and AA6061-T6	SiC	An increase in tensile strength and elongation so the joint without nanoparticles is obtained. SiC nanoparticles had a great effect on the grain refining of the SZ	[108]
AZ91	SiC	The microstructure was refined and mechanical characteristics, including hardness, ductility, and strength were improved	[109]
AZ91	Al_2_O_3_	The incorporation of Al_2_O_3_ particles can considerably improve the wear performance of this based-alloy. In addition, uniform particles distribution leads to refinement of structure and escalation of hardness	[110]
AZ91C	SiO_2_	Escalating the number of passes could enhance the distribution state of SiO_2_ reinforcing particles in the SZ, which improved the composite's mechanical strength.	[111]

**Table 4:** Advantages of the FSW/FSP technique.

Metallurgical characteristics	Mechanical Properties	Energy	Safety	Environmental	Reference
Fine and ultra-fine microstructure, Texture modification	High tensile strength and good ductility	Lower required energy for welding and favorite joint efficiency	No fumes and welding arc	Annihilation of grinding wastes and solvents for degreasing	[2,26,27,28,80,113,114]
Absence of welding and casting defects	High fracture toughness and energy absorption	Use of smaller thicknesses due to increased strength of welded material	No spatter	Not required to the shielding gas	[2,26,27,28,80,113,114]
Excellent distribution of alloying elements and precipitations and their dissolution in the matrix phase	High fatigue strength	Reduction of structure weight due to using smaller thicknesses	No spark	Not required to the surface cleaning	[2,26,27,28,80,113,114]
Good microstructural homogenization	High wear resistance	Lower consumption of fuel caused by weight reduction of structure in industries of automobile, aircraft, etc.	No radiation of ultraviolet	More decrease in production of CO_2_ gas due to lower fuel consumption	[2,26,27,28,80,113,114]

**Table 5:** Brief summary of the work carried out regarding parameters affects FSW and FSP techniques for Mg-based alloys.

Alloy	Method	Tool Material	Tool Shape	Tool Size	Parameters	Conclusions/Remarks	Reference
AA6061 and NZ30K	FSW	H13 Tool steel	Cylindrical threaded pin	Shoulder diameter: 15 mm Pin diameter: 4 mm Pin length: 3.8 mm	Rotation speed: 600 to 1500 rpm Welding speed: 60 to 120 mm/min	The highest tensile shear failure load at the rotation speed of 900 rpm and welding speed of 120 mm/min and formation of the intermetallic compound of ${\mathrm{Al}}_{3}{\mathrm{Mg}}_{2}$ in stir zone as well as occurrence of failure at the interface of the intermetallic compound layer of Mg and Al alloys	[8]
AZ31 and AZ91	FSW	H13 Tool steel	Flat shoulder with hybrid tool pin (cylindrical; having upper portion with plain profile and lower portion with threaded profile)	Pin diameter: 6 mm Tilt angle: 2.5°	Rotation speed: 700 to 1000 rpm Welding speed: 30 to 50 mm/min Shoulder diameter: 15 to 21 mm	Increment of grain size with an increase in rotation speed and shoulder diameter. Moreover, rotational speed and shoulder diameter have the most significant effect on the surface roughness and tensile strength, and flexural strength	[115]
AZ31B	FSW	High-speed steel (HSS) and Stainless steel (SS)	Taper with threaded pin	Shoulder diameter: 18 mm Pin diameter: 6 mm Pin length: 4.8 mm Axial force: 5 kN Tilt angle: 2.5°	Rotation speed: 900 to 1800 rpm Welding Speed: 40 mm/min	Excellent mechanical properties with SS tool at the rotation speed of 1120 rpm	[116]
AZ31B	FSW	HSS	Taper Cylindrical pin	Shoulder diameter: 12 mm Pin diameter: 4 mm Pin length: 4.85 mm	Rotation speed: 1800 rpm Welding speed: 50 mm/min Axial Force: 3 to 5 kN	Higher tensile strength at axial force of 5 kN	[41]
AZ31B-H24	FSW	H13 Steel	Scrolled with right hand threaded pin	Shoulder diameter: 9.5 mm Pin diameter: 3.175 mm Pin length: 1.7 mm Tilting angle: 0.5°	Rotation speed: 2000 rpm Welding speed: 5 to 30 mm/s	Increment of yield strength with an increase in welding speed. Increment of tensile strength with an increase in welding speed from 5 to 15 mm/s and remaining constant of tensile strength from 15 to 30 mm/s	[117]
Mg-9Li-1Zn	FSP	WC Cemented carbide	-	Shoulder diameter: 15 mm Pin diameter: 6 mm Pin length: 2.8 mm	Rotation speed: 30 rpm Processing speed: 10 mm/min	Getting to superplasticity of 369% and 1104% at the strain rate of $10^{-1}\;s^{-1}$ and $3.33\times 10^{-4}\;s^{-1}$, respectively at 473 K	[25]
Mg-9Al-xRE (x: Ce + La with various weight percentages)	FSP	Mo-based alloy	Threaded conical needle pin	Shoulder diameter: 16 mm The bottom diameter of pin: 6 mm Top diameter of pin: 4 mm Pin length: 4.7 mm	Rotation speed: 650 rpm Transverse speed: 25 mm/min	Increment of corrosion resistance in NaCl solution caused by fine and redistributed precipitations in α-Mg matrix	[4]
ZE41	FSP	H13 Tool steel	Tapered pin	Shoulder diameter: 15 mm Pin possessing 5 mm to an end diameter of 2 mm Pin length: 3 mm	Rotation speed: 1100 to 1800 rpm Travel speed: 16 to 50 mm/min	Higher level of grain refinement (up to 3 μm) and hardness (84.2 HV) at the rotation speed of 1400 rpm and travel speed of 25 mm/min with no imperfections	[36]
Mg-8Li-3Al-2Sn	FSP	-	Conical threaded pin	Shoulder diameter: 15 mm Pin diameter: 6.5 and 4.3 mm at the base and the tip, respectively Pin length: 8 mm	Rotation speed: 1000 rpm Travel speed: 60 mm/min	Improvement of yield strength (by 42%), ultimate tensile strength (by 20%), and elongation (by 12%) of processed alloy as compare to unprocessed alloy due to hardening effect of grain refinement of α-Mg phase and broken ${\mathrm{Li}}_{2}$MgSn phase	[118]
Mg-2Nd-0.2 Zn	FSP	-	Concave- shaped	Shoulder diameter: 20 mm Pin diameter: 6 mm Pin length: 2.3 mm Plunge depth: 1.5 mm Tool tile: 1.5°	Rotation speed: 400 and 600 rpm Transverse speed: 100 mm/min	Lower corrosion rate at the rotation speed of 400 rpm compared to the rotation speed of 600 rpm in Hank’s solution due to grain refinement, the lower volume fraction of second phases, and the stronger basal texture	[9]

**Table 6:** Evolutions of mean grain size of some alloys after FSW and FSP techniques.

Alloy	Mean Grain Size of Base Metal (BM)	Mean Grain Size of Stir Zone (SZ)	References
AZ31	75 μm	100 nm	[17,123]
AZ61	75 μm	100 nm	[43,124]
AZ91	150 μm	4 μm	[67,125]
AA1050	65 μm	0.5 μm	[126,127]
AA5052	49.4 μm	10.1 μm	[128]
AA5086	48 μm	6 μm	[61,129]

**Table 7:** Mechanical properties of Mg-based alloys before and after FSP.

Alloy	Yield Strength (MPa)	Tensile Strength (MPa)	Hardness (HV)	Elongation (%)	Method	Yield Strength (MPa)	Tensile Strength (MPa)	Hardness (HV)	Elongation (%)	Reference
Mg-6Zn	73 ± 3	130 ± 4	55 ± 6	8.9 ± 1.8	FSP	133 ± 2	280 ± 2	68 ± 4	18.6 ± 0.8	[18]
Mg-6Zn-1Y-0.5Zr	97 ± 2	200 ± 6	65 ± 3	12.5 ± 1.4	FSP	170 ± 3	310 ± 5	80 ± 2	27.7 ± 1.2	[18]
AXM541	125 ± 9	150 ± 13	53	12 ± 4	FSP	322 ± 14	361 ± 17	74	16 ± 3	[42]
Mg-Gd-Y-Zn-Zr	187 ± 5.2	249 ± 5.5	-	7.9 ± 0.6	FSP	345 ± 5.5	380 ± 9.9	-	21.5 ± 3.3	[77]
ZKX50	110.0	236.6	-	11.4	FSP	146.0	247.9	-	15.7	[133]
LZ91	149	206	-	18	FSP	140	305	-	28	[24]

## Data Availability

All data provided in the present manuscript are available to whom it may concern.
